# Proposition of a Classification of Adult Patients with Hemiparesis in Chronic Phase

**DOI:** 10.1371/journal.pone.0156726

**Published:** 2016-06-07

**Authors:** Frédéric Chantraine, Paul Filipetti, Céline Schreiber, Angélique Remacle, Elisabeth Kolanowski, Florent Moissenet

**Affiliations:** CNRFR - Rehazenter, Laboratoire d’Analyse du Mouvement et de la Posture, Luxembourg, Luxembourg; Emory University School Of Medicine, UNITED STATES

## Abstract

**Background:**

Patients who have developed hemiparesis as a result of a central nervous system lesion, often experience reduced walking capacity and worse gait quality. Although clinically, similar gait patterns have been observed, presently, no clinically driven classification has been validated to group these patients’ gait abnormalities at the level of the hip, knee and ankle joints. This study has thus intended to put forward a new gait classification for adult patients with hemiparesis in chronic phase, and to validate its discriminatory capacity.

**Methods and Findings:**

Twenty-six patients with hemiparesis were included in this observational study. Following a clinical examination, a clinical gait analysis, complemented by a video analysis, was performed whereby participants were requested to walk spontaneously on a 10m walkway. A patient’s classification was established from clinical examination data and video analysis. This classification was made up of three groups, including two sub-groups, defined with key abnormalities observed whilst walking. Statistical analysis was achieved on the basis of 25 parameters resulting from the clinical gait analysis in order to assess the discriminatory characteristic of the classification as displayed by the walking speed and kinematic parameters. Results revealed that the parameters related to the discriminant criteria of the proposed classification were all significantly different between groups and subgroups. More generally, nearly two thirds of the 25 parameters showed significant differences (p<0.05) between the groups and sub-groups. However, prior to being fully validated, this classification must still be tested on a larger number of patients, and the repeatability of inter-operator measures must be assessed.

**Conclusions:**

This classification enables patients to be grouped on the basis of key abnormalities observed whilst walking and has the advantage of being able to be used in clinical routines without necessitating complex apparatus. In the midterm, this classification may allow a decision-tree of therapies to be developed on the basis of the group in which the patient has been categorised.

## Introduction

Patients who have developed hemiparesis after a central nervous system lesion often experience reduced walking capacity and worse gait quality [1,2]. These gait disturbances are commonly due to abnormalities in gait pattern (i.e., changes at the joint kinematics level). Clinically, these abnormalities can result from one or several underlying primary and/or secondary impairments (e.g., contracture, spasticity, reduced selective motor control), but also from compensations adopted by the patient to overcome some of these problems. In order to ensure a valuable clinical management, two key objectives are 1) to identify the gait abnormalities (i.e., deviations of the kinematics from a normal gait pattern), and 2) to relate these abnormalities to impairments and/or compensation mechanisms. While it remains difficult to restore a normal gait pattern, the role of the clinician will then be to improve the walking capacities and endurance in a safe and comfortable way. In this sense, the primary gait abnormalities to be managed are those observed during the swing phase of the paretic leg, impacting foot clearance and thus increasing the risk of falls [3]. The key abnormalities that may be managed are thus: 1) a decrease in the range of hip flexion/extension [4], 2) an abnormality in knee flexion [5,6], and 3) a reduced ankle dorsiflexion [4,7].

In order to define management algorithms, but also to ease communication, the use of a classification system based on gait pattern has been proposed and used in many studies [8,9]. However, while classification has been intensively applied to cerebral palsy children [8], the literature concerning hemiparetic adults remains scarce [2,9,10]. Whatever the targeted population, two types of classification are generally described. On one hand, clinically driven classifications use a reduced number of inputs and define groups of patient from clinical knowledge [2]. One advantage of this approach is to conduct to clinically meaningful groups [8]. However, this approach is highly guided by the clinical experience of the users and can lead to differences in terms of interpretation. Furthermore, to the authors knowledge, no study demonstrated the existence of a statistical evidence validating the differences between groups in the context of hemiparetic adults. This can be illustrated by the study of de Quervain et al. [2] where the joint kinematics differences were only assumed by visual inspection, while their classification relied on these parameters and walking speed. On the other hand, statistically driven classifications use a method such as a cluster analysis to extract significantly different groups of patients in an objective manner on the basis of the analysis of many and various parameters (e.g., kinematics, kinetics, EMG) [9,10]. However, the success of these classifications relies on the parameters used in the cluster analysis, and thus on the patients cohort. They may thus lead 1) to a classification not generalisable due to a cohort only focused on a subpopulation, and/or 2) to artificial groups with a poor clinical relevance. For example, Kinsella et al. [10] only used parameters recorded on patients with a reduced knee flexion during swing phase. Consequently, patients without this abnormality are not being identified using their classification. Mulroy et al. [9] defined four groups of stroke patients using a non-hierarchical cluster analysis based on spatiotemporal and kinematic parameters. However, the parameters describing the groups obtained during acute and chronic phases were different (as well as their associated upper and lower bounds between groups). A longitudinal follow up of the patients thanks to this method may thus be difficult in a clinical routine. Furthermore, hip and knee flexions during swing phase were omitted. Consequently, ankle dorsiflexion during swing was the only considered parameter limiting the foot clearance. Again, if this classification is used in a clinical routine, it will thus not help managing abnormalities during swing, while they should be considered as primordial to avoid falls.

This study intended to put forward a gait classification for adult patients with hemiparesis. In order to keep a clinical meaning, the groups were defined from clinical knowledge, by focusing on abnormalities leading to an increase of the risk of falls (i.e., a decrease of the foot clearance). This classification was applied on patients in chronic phase and, in order to demonstrate the statistical differences between groups, an analysis of variance, based on a selection of spatiotemporal and kinematic parameters, was conducted. These parameters were obtained through a clinical gait analysis that is recognised as an objective gait assessment allowing the quantification of gait abnormalities [11].

## Materials and Methods

### Participants

Twenty-six patients (10 women, 16 men) with hemiparesis (13 left, 13 right) were included in this study within the framework of their medical supervision at the National Centre for Functional Re-education and Rehabilitation of Luxembourg (patient clinical data is supplied as S1 Table). These patients were all selected under a protocol involving the potential implantation of a functional electrical stimulation device for foot drop management. This protocol was approved by the National Research Ethics Committee of Luxembourg. All patients provided their written approval prior to their participation. Only hemiparesis associated with a central nervous system lesion have been considered (11 hemorrhagic strokes, 12 ischemic strokes, 3 other causes). Mean age, weight and height (± 1 standard deviation) of participants was 47.2 years old (±10.6), 77.8 kg (±17.4) and 171.2 cm (±7.7). Inclusion criteria were: 1) hemiparesis more than six months old (i.e., in chronic phase), 2) gait capacity of more than ten meters without technical aid, 3) no other neuro-orthopaedic pathology capable of altering the gait pattern.

### Classification

The proposed clinically driven classification consists in three groups, each divided into two sub-groups (Table 1). It was based on two assumptions. Firstly, the primary abnormalities are the ones impacting foot clearance (as well as spatio-temporal parameters [3]) and thus increasing the risk of falls. In this sense, the key abnormalities that may be managed all appear during swing phase and are: 1) a decrease in the range of hip flexion/extension [4], 2) an abnormality in knee flexion [5,6], and 3) a reduced ankle dorsiflexion [4,7]. Secondly, it was assumed that limited impairments lead to distal deficiencies, while larger impairments lead to both distal and proximal deficiencies.

**Table 1:** Discriminatory criteria of the proposed classification. Presence of kinematic abnormality or muscle strength score under 3/5 on the Medical Research Council (MRC) scale [12] is noted by X, while a possible but not essential presence is noted by (X).

	Group I		Group II		Group III	
	Ia	Ib	IIa	IIb	IIIa	IIIb
**Ankle**						
Reduced dorsiflexion in swing phase	X	X	(X)	(X)	(X)	(X)
Reduced dorsiflexion in stance phase		X				X
**Knee**						
Reduced flexion in swing phase			X	X	(X)	(X)
Genu recurvatum		X		X		X
**Hip**						
Reduced range of motion					X	X
Weak flexor muscles					X	X

The classification was thus defined through a set of discriminatory criteria composed of 1) the primary abnormalities to one or more observed joints (i.e., hip, knee and ankle) in the sagittal plane, and 2) muscle strength abnormalities observed during the clinical examination. In particular, attention was focused on propulsive muscles’ strength (i.e., hip flexor and extensor muscles and also triceps surae as ankle plantarflexor muscles).

Discriminatory criterion characterising group I is the presence of a reduced ankle dorsiflexion in swing phase (Table 1). This abnormality may be due to a foot drop or an equinus foot. In case of equinus foot, its presence in swing phase may be explained by a weakness of the tibialis anterior (and possibly to other dorsiflexor muscles) [13], and/or an increased stiffness of the triceps surae [7]. The strength of ankle plantarflexor muscles is often weak (i.e., <3/5 on the Medical Research Council (MRC) scale [12]). No kinematic abnormality is observed in the proximal joints (i.e., knee and hip joints) in swing phase. In a less regular way, group Ia may also be associated with a minor equinus foot in stance phase, which may lead to a disappearance of the foot strike by the heel. However, this equinus does not interfere with the range of ankle dorsiflexion during the stance phase.

With respect to group II, the discriminatory criterion is the presence of a reduced knee flexion in swing phase (i.e., stiff-knee) (Table 1). This abnormality is often caused by an inappropriate contraction of the rectus femoris during this phase [14]. The main impact of this abnormality is to decrease the foot elevation during swing phase, increasing the risk of falls [15,16]. The presence of compensatory phenomena (e.g., hip hiking, circumduction, vaulting) is thus possible in order to prevent the foot from dragging along the ground during the swing phase [16,17]. Once again, the strength of ankle plantarflexor muscles is often weak (i.e., <3/5 on the MRC scale). Generally, group II may also be associated with an equinus foot (with or without varus) in swing phase. Less often, the strength of the hip extensor muscles may be weak (i.e., <3/5 on the MRC scale). However, no kinematic abnormality is observed at the hip joint.

Finally, group III is characterised by the presence of a reduced range of hip motion and a weak hip flexor muscles’ strength (i.e., <3/5 on the MRC scale) (Table 1). More generally, an overall muscular weakness is observed for the entire lower limb for which spasticity is significant. On a variable basis, group III may be associated with an equinus foot (with or without varus) as well as with a reduced knee flexion in swing phase (i.e., stiff-knee).

Each group is subdivided into two sub-groups (i.e., sub-groups a and b), with genu recurvatum during the stance phase as discriminatory criteria (i.e., observable criteria for sub-groups Ib, IIb, and IIIb). This subdivision was chosen, firstly, by the frequency with which this abnormality is observed in hemiparetic patients (i.e., 40 to 68% [18]), and secondly, by the fact that this deficiency may cause articular pain and diminish gait capacity [19,20]. Genu recurvatum is often caused by the presence of an equinus foot in stance phase, disrupting the progression of the tibial segment, or a spasticity of the quadriceps [21].

### Protocol

Firstly, a clinical examination was carried out on all participants by an experienced doctor. The clinical examination included an assessment of: 1) the passive range of motion of hip, knee and ankle joints, 2) the muscle strength of the hip, knee and ankle extensor and flexor muscles on the MRC scale [12], and 3) the spasticity of the triceps surae on the Tardieu scale [22]. Note that for hip flexor muscles, strength was assessed whilst standing, the patient had to bend the hip to 90° and hold this position for at least five seconds to get 3/5 on the MRC scale. As the patient was on one foot, keeping balance with the upper limb was permitted.

Following this clinical examination, a clinical gait analysis (CGA), complemented by a video analysis, was performed, whereby participants were requested to walk spontaneously on a 10m walkway. For the CGA, six recordings were retained for each participant with a break between each trial when required. These recordings were obtained using 10 optoelectronic cameras (OQUS, Qualisys, Sweden) sampled at 100Hz, with a set of retroreflective cutaneous markers in accordance with the protocol proposed by Leardini et al. [23]. An inverse kinematic procedure based upon the use of homogeneous matrices [24] was then used to calculate kinematics under Matlab R2011b after data importation using the Biomechanical ToolKit (BTK) [25]. The video analysis was carried out under the same conditions as those used for the CGA. One recording was obtained simultaneously in the frontal and sagittal planes by two video cameras (OQUS-2c, Qualisys, Sweden).

**Fig 1:**
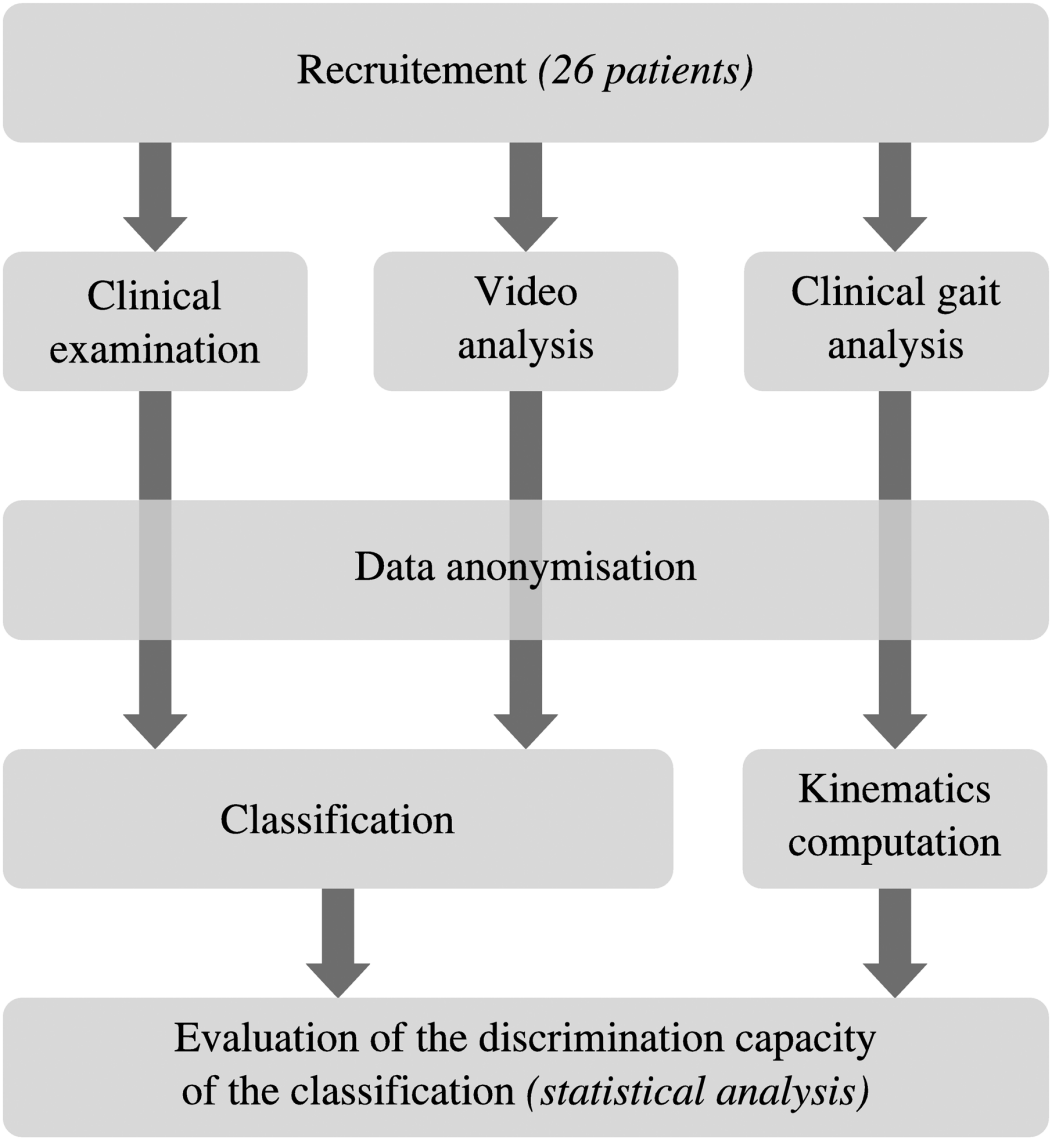
Procedure followed for this study.

Upon concluding these procedures, all of the data was made anonymous. The clinical examination results and videos were then analysed by an experienced doctor in order to establish the classification for each participant. The CGA results were finally used as a validation tool to check the discriminatory characteristic of the proposed classification on the basis of walking speed and kinematic parameters (statistical methodology described in the following paragraph). Fig 1 lists the procedure which was followed for this study.

### Statistics

**Fig 2:**
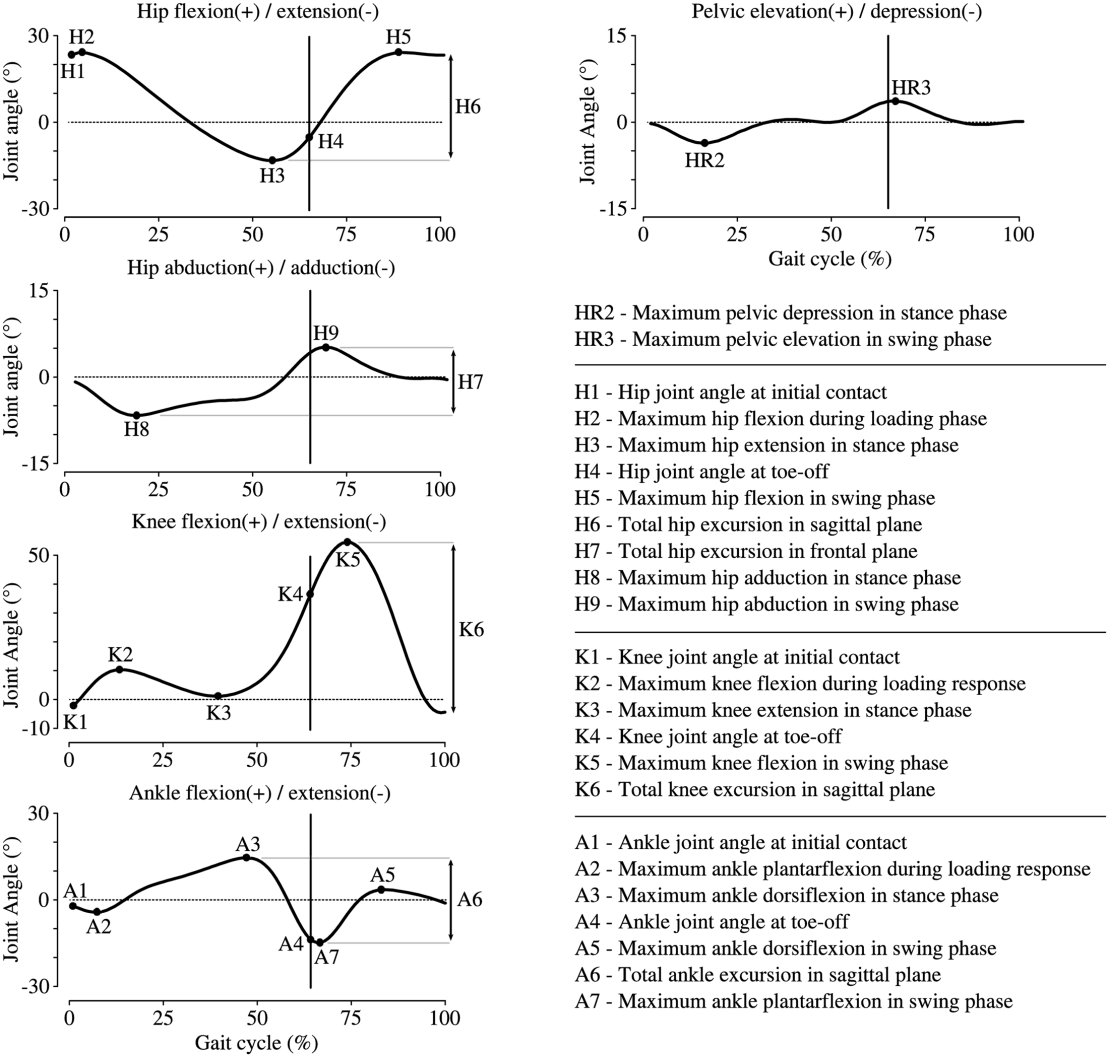
Kinematic parameters used for the analysis of variance (ANOVA).

In order to determine if the proposed classification revealed any significant differences between the groups on the basis of walking speed and kinematic parameters, an analysis of variance (i.e., ANOVA) was achieved. This analysis was based on 25 parameters composed of walking speed together with Kinsella et al.’s 24 proposed kinematic parameters [10] described in Fig 2. A subgroup of 3 parameters was identified and defined as primary parameters since they are directly related to the discriminant criteria of the proposed classification (i.e., maximum knee flexion in swing phase—K5 and total hip excursion in sagittal plane—H6 between groups, and maximum knee extension in stance phase—K3 between subgroups). While these primary parameters directly validate the discriminatory capacities of the proposed classification, other parameters highlight the other differences existing between groups.

## Results

### Classification

Among the 26 participants, only one patient was unable to be incorporated into one of the proposed classification groups. Indeed, this patient’s gait quality was excellent (i.e., spontaneous walking speed was 1.01 m.s^-1^) and the patient’s only observable abnormality was an excessive knee flexion during the stance phase.

Among the 25 remaining participants, 5 were classified in group I (i.e., 1 patient in sub-group Ia, 4 patients in sub-group Ib), 15 patients in group II (i.e., 7 patients in sub-group IIa, 8 patients in sub-group IIb), and 5 in group III (i.e., 1 patient in sub-group IIIa, 4 patients in sub-group IIIb).

### Differences between groups

Concerning the primary parameters related to the discriminant criteria between groups, results show that the maximum knee flexion in swing phase (K5) was always significantly different between groups (Table 2), as well as the total hip excursion in sagittal plane (H6). More generally, 16 of the 25 assessed parameters revealed a significant level of difference between at least two groups (sub-groups a and b combined) (Table 2). These differences are set out here below.

**Table 2:** Mean and standard deviation of the parameters assessed for each group (GI—Group I, GII—Group II, GIII—Group III) and results of the analysis of variance (ANOVA). Primary parameters related to the discriminant criteria between groups are in bold.

	GI (n = 5)	GII (n = 15)	GIII (n = 5)	ANOVA		
				GI vs. GII (p value)	GI vs. GIII (p value)	GII vs. GIII (p value)
**Spatiotemporal parameters**						
Walking speed (m.s^-1^)	*0*.*86±0*.*19*	*0*.*68±0*.*22*	*0*.*49*±*0*.*25*	*<0*.*001*	*<0*.*001*	*<0*.*001*
**Pelvis kinematics**						
HR2 (°)	*-2*.*2±1*.*9*	*-1*.*1±3*.*4*	*0*.*2*±*2*.*3*	*NS*	*<0*.*001*	*NS*
HR3 (°)	*3*.*3±1*.*2*	*6*.*9±3*.*1*	*9*.*8*±*4*.*1*	*<0*.*001*	*<0*.*001*	*<0*.*001*
**Hip kinematics**						
H1 (°)	*14*.*0±7*.*6*	*16*.*3±8*.*6*	*16*.*6*±*10*.*0*	*NS*	*NS*	*NS*
H2 (°)	*14*.*2±7*.*7*	*17*.*7±9*.*3*	*16*.*8*±*9*.*9*	*NS*	*NS*	*NS*
H3 (°)	*-17*.*8±14*.*8*	*-10*.*8±8*.*7*	*-0*.*6*±*3*.*9*	*<0*.*01*	*<0*.*001*	*<0*.*001*
H4 (°)	*-5*.*1±11*.*9*	*-1*.*9±7*.*5*	*4*.*9*±*5*.*1*	*NS*	*<0*.*001*	*<0*.*001*
H5 (°)	*20*.*1±8*.*6*	*21*.*5±8*.*0*	*23*.*2*±*11*.*0*	*NS*	*NS*	*NS*
**H6 (°)**	***38*.*1±8*.*8***	***33*.*2±6*.*7***	***23*.*8*±*10*.*8***	***<0*.*01***	***<0*.*001***	***<0*.*001***
H7 (°)	*10*.*9±1*.*9*	*10*.*3±3*.*1*	*8*.*8±4*.*0*	*NS*	*NS*	*NS*
H8 (°)	*-8*.*2±3*.*0*	*-6*.*3±4*.*4*	*-5*.*6±4*.*0*	*NS*	*NS*	*NS*
H9 (°)	*2*.*7±2*.*4*	*4*.*0±4*.*1*	*3*.*3±2*.*0*	*NS*	*NS*	*NS*
**Knee kinematics**						
K1 (°)	*-4*.*6±10*.*5*	*4*.*8±6*.*3*	*2*.*0*±*4*.*7*	*<0*.*001*	*<0*.*01*	*<0*.*05*
K2 (°)	*1*.*5±13*.*5*	*11*.*8±9*.*2*	*3*.*4*±*5*.*3*	*<0*.*001*	*NS*	*<0*.*001*
K3 (°)	*-13*.*1±14*.*7*	*-5*.*0±11*.*1*	*-5*.*5*±*4*.*9*	*<0*.*01*	*<0*.*05*	*NS*
K4 (°)	*23*.*3±10*.*0*	*16*.*9±9*.*2*	*9*.*3*±*5*.*0*	*<0*.*01*	*<0*.*001*	*<0*.*001*
**K5 (°)**	***39*.*9±10*.*5***	***26*.*0±10*.*7***	***13*.*6*±*7*.*5***	***<0*.*001***	***<0*.*001***	***<0*.*001***
K6 (°)	*53*.*2±13*.*3*	*33*.*9±12*.*1*	*21*.*7*±*9*.*2*	*<0*.*001*	*<0*.*001*	*<0*.*001*
**Ankle kinematics**						
A1 (°)	*-14*.*7±2*.*7*	*-11*.*7±7*.*9*	*-11*.*4*±*6*.*6*	*NS*	*<0*.*05*	*NS*
A2 (°)	*-14*.*9±2*.*7*	*-11*.*9±7*.*8*	*-12*.*2*±*7*.*5*	*NS*	*NS*	*NS*
A3 (°)	*9*.*9±5*.*0*	*10*.*2±3*.*6*	*7*.*3*±*7*.*8*	*NS*	*NS*	*<0*.*05*
A4 (°)	*-6*.*3±8*.*3*	*-8*.*2±9*.*4*	*-6*.*9*±*6*.*2*	*NS*	*NS*	*NS*
A5 (°)	*-5*.*5±7*.*8*	*-6*.*7±10*.*0*	*-6*.*6*±*7*.*9*	*NS*	*NS*	*NS*
A6 (°)	*27*.*8±6*.*4*	*27*.*6±12*.*3*	*21*.*4*±*10*.*6*	*NS*	*<0*.*05*	*<0*.*05*
A7 (°)	*-17*.*3±3*.*3*	*-16*.*8±13*.*0*	*-12*.*8*±*6*.*6*	*NS*	*<0*.*01*	*NS*

#### Group I versus Groups II and III

Walking speed of group I was significantly greater than in groups II and III (+0.18m.s^-1^ and +0.37m.s^-1^ respectively). At the pelvis level, the maximum depression in stance phase (HR2) was significantly reduced for group III, whereas the maximum elevation in swing phase (HR3) was significantly amplified for groups II and III (+3.6° and +6.5° respectively). At the hip level, the maximum extension in stance phase (H3) was significantly reduced for groups II and III (-7.0° and -17.2° respectively), leading to a decreased excursion in sagittal plane (H6) (-4.9° and -14.3° respectively). For these two parameters, the observed reduction was greater for group III and was complemented by a major increased of the hip joint angle at toe-off (H4). At the knee level, all the observed parameters revealed a significant modification except for the maximum flexion during loading phase (K2) between groups I and III. In particular, we have noted a significant reduction of the maximum flexion in swing phase (K5) for groups II and III (-13.9° and -26.3° respectively), which was more marked for group III. At the ankle level, a dorsiflexion abnormality in swing phase (i.e., negative A5 criteria) was observed in the three groups. Only three parameters were significantly different between groups I and III (i.e., joint angle at initial contact (A1), maximum plantarflexion in swing phase (A7) and total joint excursion in sagittal plane (A6)).

#### Group II versus Group III

On the whole, the same major differences were observed between group II versus group III and those observed between group I versus groups II and III with the exception of the appearance of a significant reduction in maximum ankle dorsiflexion in stance phase (A3) (-2.9°). More specifically, at the pelvis level, elevation (HR3) was significantly increased for group III (+2.9°). At the hip level, the maximum extension in stance phase (H3) was significantly reduced for group III (-10.2°), leading to a decreased total joint excursion in sagittal plane (H6) (-9.4°). At the knee level, all the observed parameters revealed a significant modification except for the maximum extension in stance phase (K3) between groups II and III. In particular, we have noted a reduction of the maximum flexion in swing phase (K5) for group III (-12.4°). At the ankle level, only two parameters revealed any sizeable differences (i.e., maximum dorsiflexion in stance phase (A3), and total joint excursion in sagittal plane (A6)).

### Differences between subgroups

Only group II contained sufficient participants to enable the differences between the sub-groups a (i.e., without genu recurvatum) and b (i.e., with genu recurvatum) to be assessed. Of the 15 listed participants in group II, 7 have been classified in the sub-group IIa and 8 in the sub-group IIb. Observed differences in relation to walking speed and the knee kinematic parameters are reported in Table 3.

**Table 3:** Mean and standard deviation of the knee kinematic parameters for sub-groups IIa (GIIa) and IIb (GIIb) and results of the analysis of variance (ANOVA). Primary parameters related to the discriminant criteria between subgroups are in bold.

	GIIa (n = 7)	GIIb (n = 8)	ANOVA
			GIIa vs. GIIb (p value)
**Spatiotemporal parameters**			
Walking speed (m.s^-1^)	*0*.*66±0*.*29*	*0*.*70±0*.*13*	*NS*
**Knee kinematics**			
K1 (°)	*6*.*9±6*.*0*	*3*.*0±6*.*1*	*< 0*.*001*
K2 (°)	*15*.*4±8*.*5*	*8*.*7±8*.*7*	*< 0*.*001*
**K3 (°)**	***1*.*5±7*.*6***	***-10*.*5±10*.*6***	***< 0*.*001***
K4 (°)	*19*.*8±7*.*6*	*14*.*3±9*.*7*	*< 0*.*05*
K5 (°)	*27*.*4±13*.*7*	*24*.*9±7*.*2*	*NS*
K6 (°)	*29*.*3±15*.*4*	*37*.*7±6*.*2*	*< 0*.*001*

At the knee level, all parameters revealed a significant difference between sub-groups IIa and IIb with the exception of the maximum flexion in swing phase (K5). However, the key difference was related to the primary parameter, the maximum extension in stance phase (K3), presenting an increased extension of +12° for sub-group IIb. No significant difference was observed in relation to walking speed.

## Discussion

As a result of a central nervous system lesion, numerous patients retain hemiparesis, driving to gait abnormalities, which limit their autonomy. In such patients, gait patterns are often similar, leading one to believe that a clinical classification of their gait may be possible. On the basis of three abnormalities retained as mainly disruptive to gait quality for this patient population, a classification system was implemented. This classification consists in three groups, characterised by the abnormalities’ type, starting from the most distal features (i.e., group I) and moving towards features which are both distal and proximal (i.e., group III). Each group is composed of two sub-groups corresponding to the presence (e.g., group Ib) or not (e.g., group Ia) of a genu recurvatum.

Obtained results demonstrate, firstly, the efficiency of the proposed classification to regroup patients on the basis of their gait pattern. Indeed, of the 26 patients included in the study, only one could not be included in a group. However, this patient displayed good walking speed and largely preserved gait quality. Among the remaining 25 patients, the distribution was not uniform throughout the groups and sub-groups. Whereas groups I and III received a small number of patients, group II received three times as many patients. This observation may be explained by the fact that patients in group I displayed minor gait abnormalities, and are thus barely monitored at a rehabilitation centre. Furthermore, patients that could be classified in group III often walk with technical aid (e.g., cane), and thus, many do not fall within the inclusion criteria of this study. Distribution on the basis of a genu recurvatum presence was largely balanced (i.e., 64% of patients with genu recurvatum) and corresponds to the values reported in the literature [18]. Comparison of the groups obtained with a set of key parameters [10] revealed an excellent group differentiation, both in relation to walking speed and kinematic parameters. With respect to walking speed, the difference between groups was all greater than the minimal clinically important difference (MCID) for this population (i.e., 0.10m.s^-1^) [26]. With respect to kinematic parameters, results showed that all the primary parameters, linked to the discriminant criteria of the classification, were significantly different between groups and subgroups. This result demonstrates the excellent discriminatory capacity of the proposed classification. Moreover, the overall assessed parameters were significantly different between at least two groups for 64% of them. This rate is much higher than the one obtained by Kinsella et al. [10] (i.e., 44%) for the same parameters, which shows the clear differences existing between groups and subgroups in terms of kinematic patterns. Thus, at the pelvis level, an increase of the ipsilateral hemipelvis elevation appeared in swing phase for groups II and III, suggesting a compensatory phenomenon facilitating the foot clearance [16,17]. At the hip level, group III displayed a barely noticeable extension in stance phase, as opposed to groups I and II, for whom this extension was retained. This result, also observed by Kinsella et al. [10] and Mulroy et al. [9], has been explained by these authors as a weakness of the hip extensor muscles. However, motor selectivity disorders are often observed after a central nervous system lesion, leading to a temporary disruption of the activation of the hip extensor and flexor muscles (i.e., potential agonist/antagonist co-contractions of these muscles), reducing the hip range of motion [4]. This type of disorder is more frequent in patients displaying extensive gait abnormalities (i.e., group III). At the knee level, groups II and III displayed a clear decrease in the flexion peak in swing phase, which may be related to a knee stiffness resulting from inappropriate quadriceps activity and, in particular, rectus femoris (e.g., due to spasticity) [15,17], a decreased ankle plantarflexion, or a reduced hip flexion. Differences were less marked at the ankle level. This may notably be explained by the fact that 92% of participants displayed dorsiflexion abnormalities in swing phase associated with a foot drop or equinus foot. This percentage may appear significant when compared to the literature (i.e., Verdie et al. [27] reported approximately 18% of equinus foot and/or varus following a stroke), but must be considered in light of the fact that only hemiparetic adult patients with gait disorders were included in our study, as opposed to earlier studies. However, this factor is not restrictive for the use of the proposed classification, given that this abnormality is not necessarily represented in groups II and III (Table 1). Finally, the analysis of sub-groups IIa and IIb revealed a significant increase in maximum knee extension in stance phase, confirming the presence of a genu recurvatum.

Despite the strong discriminatory character of this classification, the groups of the classification system may not easily be associated with the groups proposed by Mulroy et al. [9] and Kinsella et al. [10]. Firstly, most of the patients included in these studies walked with a walking speed being less than 0.50m.s^-1^, with the exception of group I FAST described by Mulroy et al. [9]. Secondly, kinematic abnormalities, on which our classification system is based by their clinical importance, were not or barely retained as differentiating factors in these two earlier studies. As such, only the decrease in the range of hip flexion/extension was seen as being significantly different between groups 1 and 2 by Kinsella et al. [10], but not by Mulroy et al. [9]. Moreover, knee flexion abnormality in swing phase was not seen as significantly different in any of the two classifications. Finally, decreased ankle dorsiflexion in swing phase revealed a difference between Mulroy et al’s groups [9], without being qualified as a differentiating factor in their study. Nevertheless, it must be noticed that the genu recurvatum’s phenomenon (i.e., differentiation of the sub-groups a and b in our classification system), was observed as being significantly different between the groups of these two classification systems. But since the literature mentioned that approximately 50% of patients display this abnormality [18], and it is thus obvious to find it in each classification system. Finally, group III of our classification system may possibly be associated with the Slow gait velocity group described by De Quervain et al. [2]. Indeed, this group also corresponds to patients suffering abnormalities at ankle, knee and hip joints, with key, noticeable abnormalities at the hip. In their group, we have also observed the possible presence of reduced knee flexion in swing phase. However, patients of groups I and II of our classification are not identifiable with their classification system.

This study has certain limitations. Firstly, prior to a clinical use of this classification, a study integrating a greater number of patients must be carried out. Such a study would enable the discriminatory character of the proposed classification to be strengthened, but also to ensure that most of adult patients with hemiparesis in chronic phase displaying abnormalities disrupting the foot clearance, and thus increasing fall risks, are integrated into the study. Secondly, out of concern for patient longitudinal follow, this classification system may be applied to patients in acute phase. Indeed, even if the object of our classification was to propose midterm therapies associated with chronic patients, the progress of patients between acute and chronic phases may bring to light clinically interesting factors. Thirdly, inter-operator repeatability tests must be considered. Indeed, categorisation change from a group or a sub-group to another in our classification is based upon 1) the identification of a knee flexion abnormality in swing phase (i.e., border between groups I and II), 2) on the observation of a reduced hip range of motion in sagittal plane associated with a significant weakness of the hip flexor muscles (i.e., border between groups I/II and III) and 3) on the presence of a genu recurvatum (i.e., border between sub-groups a and b). However, the first two items may raise concerns since they are only assessed on the basis of a clinical examination and a video analysis. With respect to the adult patients’ knee flexion abnormalities in swing phase (i.e., stiff-knee), several definitions are represented in the literature [15,16,28,29] and are often inappropriate for a visual assessment. With respect to the hip flexor muscles weakness, the difficulty notably arises out of the standardisation of strength assessment of the hip flexor muscles in hemiparetic patients. As set out previously, such a strength has been assessed in our study in a standing position, the patient having to maintain a 90 degrees hip flexion during five seconds in order to obtain a score of 3/5 on the MRC scale. We believe that this approximation is precise enough for the assessment to be carried out by any clinician without any difficulty. However, another problem arises out of the definition of the minimum strength required for a satisfactory hip flexion in swing phase (i.e., key characteristic of group III). Indeed, the border of 3/5 on the MRC scale remains arbitrary. In this regard, Roche et al. [3] found no correlation between the assessment of the maximum hip flexor muscles strength in a lying down position and the hip flexion peak in swing phase. However, Hyngstrom et al. [30] established a correlation between the sub-maximal moment of the hip flexor muscles, measured by an isometric contraction in a standing position, the walking speed and the patient’s balance following a stroke. Last but not least, only sagittal and single joint abnormalities are used to group patients in our classification. However, several studies highlighted potential synergies across planes and joints [31–34]. These synergies are, in particular, related to muscle synergies as well as joint coordination, and reflect the underlying neurological and mechanical functions [35]. Consequently, interpretations may not be possible or biased when analysing independently each variable. However, even if these synergies can not be analysed through the simple protocol used for our classification (i.e., live or video assessment and clinical examination), clinical knowledge can relate several joint abnormalities. For example, as discussed previously, a reduced knee flexion during swing may be related to a reduced hip flexion or a reduced plantarflexion, as well as genu recurvatum caused by the presence of an equinus foot in stance phase. Anyway, a potential improvement may be to extend the present classification to other planes (i.e., frontal and transversal), accessible through video assessments.

In conclusion, this study puts forward a classification of adult patients with hemiparesis in chronic phase, based on abnormal gait patterns clinically identified as fall risk facilitators. This classification appears quite discriminating and has the advantage of being able to be used in a clinical routine without necessitating complex apparatus. In the midterm, this classification may allow a decision-tree of therapies to be developed on the basis of the group in which the patient has been categorised. For that, the causes of the abnormalities observed with the proposed classification may be first identified and associated to impairments observed during the clinical examination. For example, a reduced knee flexion may be the consequence of 1) a decreased ankle plantarflexion, 2) a spasticity of the quadriceps, or 3) a reduced hip flexion. Depending on the cause, an adapted treatment may be defined.

For group I, preferred therapies should therefore treat a limited ankle dorsiflexion in stance phase (e.g. injections of botulinum toxin in the triceps surae [36], AFO braces [37], use of a functional electrical stimulation device [38]). For group II, the reduced knee flexion may be managed, if related to the spasticity of the rectus femoris, by planning injections of botulinum toxin in the rectus femoris [14], or a partial selective neurotomy of the motor branch of the rectus femoris nerve [39]. Finally, for group III, other than possible cares for ankle and knee abnormalities, an efficient treatment capable of improving the hip range of motion does not exist, and thus remains to be developed.

## Supporting Information

S1 Table Clinical data of all participants (S1_Table.docx). (DOCX)

S2 Table Clinical examination results of all participants (S2_Table.xlsx). (XLS)
