# Supplementary material for: Proposition of a Classification of Adult Patients with Hemiparesis in Chronic Phase
Source: PLoS One. 2016 Jun 7;11(6):e0156726. doi: 10.1371/journal.pone.0156726 (PMC4896619; doi:10.1371/journal.pone.0156726)
Supplement: S1 Table — (DOCX) [file pone.0156726.s001.docx]

| **Patient ID** | **Gender** | | **Age (years)** | **Height (cm)** | **Weight (kg)** | **Source of hemiparesia** | | | **Affected side** | | **Cerebrovascular accident distance /assessment (months)** |
| --- | --- | --- | --- | --- | --- | --- | --- | --- | --- | --- | --- |
|  | **Woman** | **Man** |  |  |  | **Hemorrhagic stroke** | **Ischemic stroke** | **Other** | **Right** | **Left** |  |
| Patient01 |  | X | 49 | 177.5 | 88.8 |  | X |  |  | X | 32 |
| Patient02 |  | X | 15 | 178.0 | 60.0 | X |  |  | X |  | 22 |
| Patient03 | X |  | 53 | 175.0 | 58.0 |  | X |  |  | X | 35 |
| Patient04 | X |  | 37 | 181.0 | 125.0 |  | X |  | X |  | 13 |
| Patient05 |  | X | 53 | 166.5 | 105.0 |  | X |  |  | X | 25 |
| Patient06 |  | X | 53 | 184.0 | 94.0 | X |  |  | X |  | 11 |
| Patient07 |  | X | 58 | 171.0 | 84.0 | X |  |  | X |  | 13 |
| Patient08 | X |  | 45 | 160.0 | 64.0 |  | X |  | X |  | 8 |
| Patient09 | X |  | 50 | 164.0 | 72.0 | X |  |  |  | X | 67 |
| Patient10 |  | X | 32 | 167.5 | 79.0 |  | X |  |  | X | 30 |
| Patient11 |  | X | 43 | 176.0 | 81.0 | X |  |  |  | X | 129 |
| Patient12 | X |  | 36 | 164.0 | 63.3 | X |  |  | X |  | 66 |
| Patient13 | X |  | 35 | 166.0 | 54.0 | X |  |  | X |  | 7 |
| Patient14 | X |  | 56 | 166.0 | 58.9 | X |  |  | X |  | 71 |
| Patient15 |  | X | 66 | 171.5 | 75.0 |  | X |  |  | X | 12 |
| Patient16 |  | X | 36 | 167.0 | 68.9 |  |  | X | X |  | 112 |
| Patient17 | X |  | 52 | 154.0 | 66.2 | X |  |  |  | X | 57 |
| Patient18 |  | X | 55 | 181.5 | 75.6 |  | X |  | X |  | 7 |
| Patient19 |  | X | 53 | 180.0 | 112.0 |  | X |  |  | X | 39 |
| Patient20 |  | X | 52 | 171.5 | 88.0 |  |  | X | X |  | 22 |
| Patient21 | X |  | 47 | 172.0 | 60.0 |  | X |  | X |  | 71 |
| Patient22 |  | X | 50 | 168.0 | 68.6 | X |  |  |  | X | 10 |
| Patient23 |  | X | 40 | 180.0 | 81.5 |  |  | X |  | X | 216 |
| Patient24 | X |  | 55 | 167.0 | 73.0 |  | X |  | X |  | 13 |
| Patient25 |  | X | 58 | 162.0 | 76.5 |  | X |  |  | X | 13 |
| Patient26 |  | X | 47 | 181.0 | 90.0 | X |  |  |  | X | 14 |
